# Infant with Groin Swelling

**DOI:** 10.5811/cpcem2022.6.56426

**Published:** 2022-08-08

**Authors:** Nisha Polavarapu, Brendan Kilbane

**Affiliations:** * Hackensack Meridian School of Medicine, Hackensack University Medical Center, Department of Pediatric Emergency Medicine, Hackensack, New Jersey; † Rainbow Babies and Children’s Hospital, Department of Pediatric Emergency Medicine, Cleveland, Ohio

**Keywords:** hernia, groin, ovary, infant, ultrasound

## Abstract

**Case Presentation:**

A 21-day-old female presented to the pediatric emergency department with swelling of the left groin. Physical examination revealed a soft, nontender abdomen and a two-centimeter firm and fixed mass on the left aspect of her mons pubis. Point-of-care ultrasound revealed a left inguinal hernia with incarcerated ovary.

**Discussion:**

Inguinal hernias are common in the pediatric population. In female patients, particularly those less than one year old, inguinal hernias most frequently contain an ovary rather than bowel; so they require careful evaluation to protect future reproductive function.

## CASE PRESENTATION

A 21-day-old female presented to the pediatric emergency department with swelling of the left groin, which was first noticed while her family changed her diaper the morning of presentation. They denied any redness or warmth over the site but felt that she was fussier than normal overnight. They reported that she fed normally that morning and had her normal number of wet diapers. The patient was otherwise healthy and born at 37 weeks gestation without complications. On physical examination, the patient was well appearing with a soft, nontender abdomen and an irreducible two-centimeter (cm) firm and fixed mass on the left aspect of her mons pubis that was tender to palpation.

**Image 1:**
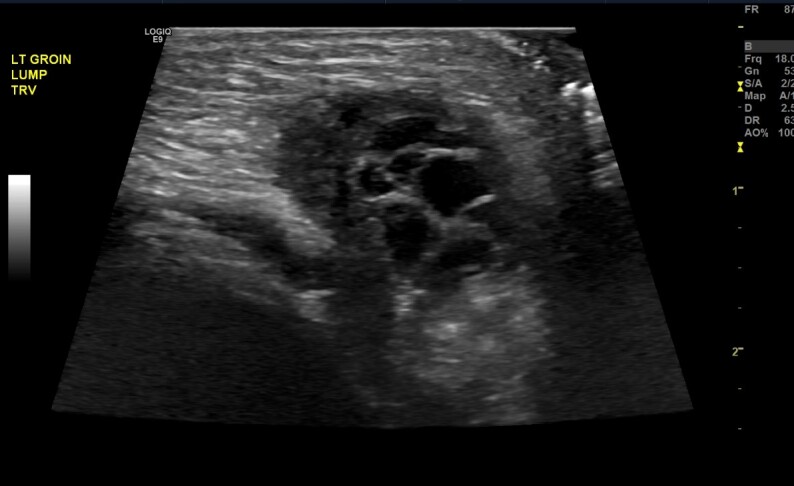
Ultrasonography (transverse view) showing an incarcerated ovary (arrow) within an inguinal hernia.

**Image 2:**
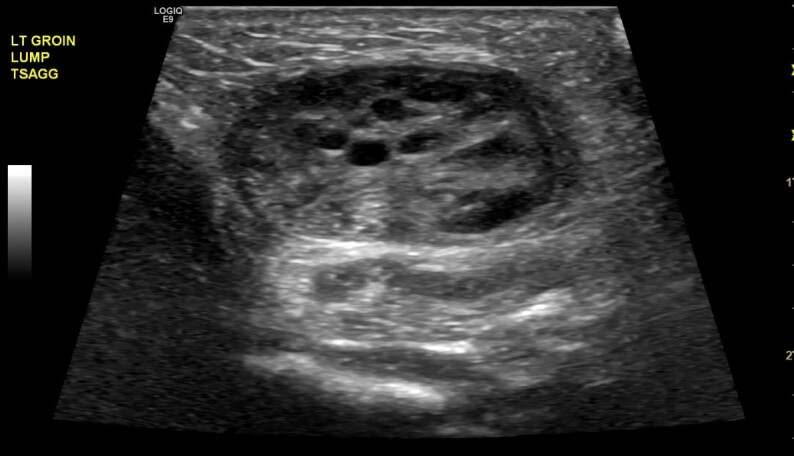
Ultrasonography (sagittal view) showing an incarcerated ovary (arrow) within an inguinal hernia.

**Image 3:**
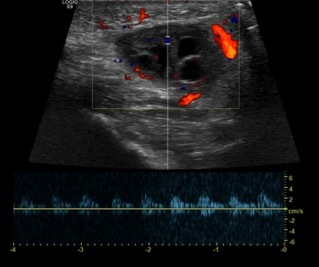
Ultrasonography with Doppler showing an incarcerated ovary (arrow) within an inguinal hernia.

A point-of-care ultrasound was performed and showed a two-cm mass with follicles. A confirmatory formal ultrasound was subsequently obtained, which showed a 2.1 × 1.1 × 2.1 cm mass in the left perineal region with subcentimeter follicles and flow demonstration, which confirmed the diagnosis of an inguinal hernia with incarcerated ovary (Images 1–3). Pediatric surgery was consulted, and the patient was taken to the operating room for left inguinal hernia repair with reduction of an incarcerated ovary.

## DISCUSSION

Inguinal hernias in the pediatric population are more commonly seen in premature infants, in male infants, and in those less than one year old.^1^ In female patients, particularly those less than a year old, inguinal hernias most frequently contain an ovary rather than bowel; so they require careful evaluation to protect future reproductive function.^2^ Prompt ultrasound is important in females if the mass is not easily reducible, as forceful reduction of the mass could result in torsion.^3^ Ultrasound will differentiate the contents of the hernia sac and determine whether appropriate blood flow is present if an ovary is involved.^4^ An irreducible ovary is at higher risk for ovarian torsion, given alteration of anatomy, and should be treated as a surgical emergency.^5^

**CPC-EM Capsule**

What do we already know about this clinical entity?

*Inguinal masses are a common chief complaint in infants presenting for emergency care. Majority of these are inguinal hernias and may contain bowel*.

What is the major impact of the image(s)?

*These images demonstrate an inguinal hernia in a female containing an ovary rather than bowel*.

How might this improve emergency medicine practice?

*Point-of-care ultrasound to identify the contents of an inguinal mass will improve and expedite patient care and management*.
